# Predictors of Social Frailty and Depression in Brazilian Patients With Chronic Kidney Disease: A Cross‐Sectional Study

**DOI:** 10.1111/ggi.70317

**Published:** 2026-01-01

**Authors:** Diana Gabriela Mendes dos Santos, Luma Geraigire, Layana Giselly Silva Ferreira, Cleanderson Costa da Silva, Ana Carolina de Souza Dinardi, Marisa Silvana Zazzetta, Fabiana de Souza Orlandi

**Affiliations:** ^1^ Department of Nursing Federal University of São Carlos São Carlos Brazil; ^2^ Department of Gerontology Federal University of São Carlos São Carlos Brazil

**Keywords:** chronic, depression, frailty, renal insufficiency, social frailty, social support

## Abstract

**Background:**

Social frailty is linked to adverse health outcomes, including depression, especially in older and chronically ill individuals. This study aimed to assess the prevalence and associated factors of social frailty in Brazilian CKD patients undergoing hemodialysis or kidney transplantation and to examine its predictive role in the development of depressive symptoms.

**Methods:**

This cross‐sectional, correlational, and comparative study included 284 patients with CKD from São Paulo, Brazil. Data were collected using validated instruments: HALFT Social Frailty Scale, Patient Health Questionnaire‐9 (PHQ‐9), and Medical Outcomes Study Social Support Scale (MOS). Two multiple linear regression (MLR) models were used to assess predictors of social frailty and depressive symptoms, adjusting for sociodemographic and clinical variables.

**Results:**

Social frailty was significantly more prevalent among HD patients (51.2%) compared to TX patients (24%). Social support was negatively associated with social frailty (β = −0.40; *p* < 0.001). Other predictors of greater social frailty included lower income (β = 0.51 for ≤ 1 minimum wage), number of medications (β = 0.11), and lower education (β = −0.13). In turn, social frailty was the strongest predictor of depressive symptoms (β = 0.60; *p* < 0.001). TX status and male sex were associated with lower depression scores.

**Conclusion:**

Social frailty is highly prevalent in CKD patients, particularly those undergoing HD, and strongly predicts depressive symptoms. Strategies to enhance social support and reduce socioeconomic vulnerability may help mitigate mental health burdens in this population.

## 1 Introduction

Social frailty, influenced by factors such as social networks, social support, and access to resources, plays a crucial role in promoting quality of life and well‐being, particularly in older adults [1]. Bunt's [2] model highlights four key factors that shape social frailty: social needs, social resources (including support and networks), social behaviors, and general resources like housing, education, and financial status. Research on social frailty has been notably prominent in the Asia‐Pacific region due to the growing elderly population and their complex biopsychosocial needs [3, 4, 5, 6, 7, 8].

Several studies have examined the impact of social frailty on a range of negative health outcomes [6, 7, 8, 9, 10]. Hamid et al. [7] identified six domains contributing to social frailty: social networks, activities, support, financial difficulties, social roles, and sociodemographic factors such as education and housing. Their findings linked social frailty to negative outcomes, including mortality, physical frailty, mobility issues, and nutritional disorders. Similarly, Li et al. [6] emphasized that social frailty in older adults predicts adverse outcomes like mortality, disability, and depression. A study by Sun et al. [9] demonstrated that social frailty, alongside sarcopenia and oral frailty, was strongly associated with depressive symptoms among Chinese adults aged 60 and over. These studies, among others, confirm the significant association between social frailty and depression. In a scoping review, Qi and Li [10] further confirmed the significant association between social frailty and depressive symptoms.

In patients with chronic kidney disease (CKD), social frailty becomes even more relevant, as these individuals often experience early onset of aging‐related conditions. Physical frailty, common in this population, is associated with an increased risk of hospitalization, falls, polypharmacy, and cognitive and functional decline [5, 10, 11, 12]. Among kidney transplant recipients, frailty is linked to delayed graft function, prolonged hospital stays, and higher mortality. Usui et al. [3] found that 59.5% of a sample of 158 patients undergoing hemodialysis (HD) presented social frailty, which was associated with reduced gait speed, leg strength, and oxygen consumption. Interestingly, patients who were neither physically nor socially frail maintained adequate physical function, whereas those with both forms of frailty showed significant impairment.

Despite the growing body of literature on social frailty in chronic populations, the direct relationship between social frailty and depression in CKD patients, especially in Brazil, remains underexplored. This gap is particularly concerning given the rising prevalence of CKD in Brazil. Between 2019 and 2023, primary care visits for CKD increased by 152.81%, particularly in the Southeast and South regions [13]. Additionally, the 2019 National Health Survey [14] found that 1.5% of Brazilian adults reported a diagnosis of chronic kidney failure, with a higher prevalence among older adults. In 2023, Brazil saw a rise in kidney transplants (TX), with more than 4500 surgeries, with kidneys accounting for 66.72% of all transplanted organs [15].

Given these trends, CKD patients in Brazil face significant challenges, including navigating healthcare services, understanding disease progression, and managing the psychosocial burden associated with treatment [16, 17]. The need for psychosocial support is particularly urgent in advanced stages of CKD, yet existing support systems are often inadequate [18]. As a result, addressing psychosocial factors, such as social frailty, is crucial in managing these patients' health.

Although social frailty is recognized as a risk factor for the development of depressive symptoms, few studies have specifically examined this relationship in patients with CKD. This study aims to fill this gap by assessing the prevalence of social frailty and identifying its associated factors in Brazilian CKD patients undergoing HD and TX. Additionally, it seeks to examine the predictive role of social frailty on depressive symptoms in this population, considering the influence of socioeconomic and clinical variables. The hypothesis is that lower levels of social support are associated with higher levels of social frailty, which, in turn, may predict more severe depressive symptoms.

## 2 Methods

### 2.1 Design and Setting

This was a cross‐sectional, correlational, and comparative study with a quantitative approach, conducted with two groups of patients: 80 undergoing hemodialysis (HD) and 204 kidney transplant recipients (TX). Data were collected from June to November 2019 (HD group) and from June to November 2021 (TX group), in cities located in the interior of the state of São Paulo, Brazil.

### 2.2 Study Size

The sample size was calculated based on the estimation of the correlation coefficient between two numerical or ordinal variables, using a significance level of 5% (α = 0.05) and a statistical power of 80% (β = 0.20), following the method proposed by Hulley et al. [19]. To detect a correlation of *r* = 0.20 between social frailty and physical frailty (as well as other variables of interest) among hemodialysis and kidney transplant patients, a minimum sample size of 194 participants was determined to be adequate.

### 2.3 Participants

A total of 1275 individuals were assessed for eligibility, of whom 913 declined to participate and 140 were excluded. The final sample consisted of 80 HD and 204 TX patients. Inclusion criteria for the HD group included being 18 years or older, diagnosed with chronic kidney disease (CKD), undergoing hemodialysis, and having preserved oral communication. Patients with a documented diagnosis of dementia were excluded. For the TX group, eligible participants were those who had undergone kidney transplantation at least 1 month prior, with preserved oral communication. Exclusion criteria included illiteracy and lack of internet access.

Participants were recruited in person for the HD group and by phone for the TX group. Those who met the eligibility criteria and agreed to participate were included in the study. HD patients signed a printed informed consent form, while TX patients provided consent electronically. The study was approved by the Research Ethics Committee of the Federal University of São Carlos. This study may be subject to selection bias due to the exclusion of illiterate participants in the transplant group, and to information bias arising from self‐reported measures of depression and social support.

### 2.4 Variables and Instruments

#### 2.4.1 Dependent Variables (Outcomes)

- **Social Frailty (in Model 1)**: Treated as a continuous and categorical variable, measured using the *HALFT Social Frailty Scale*, which ranges from 0 to 5. Scores were categorized as: 0 (non‐frail), 1–2 (pre‐frail), and ≥ 3 (socially frail). The *HALFT* was created by Ma, Sun, and Tang [20] in China and validated by Damasceno et al. [21] in Brazil. The authors named the scale HALFT, which refers to an acronym for five components: Help, pArticipation, Loneliness, Financial, and Talk.
- **Depression (in Model 2)**: Assessed using the *Patient Health Questionnaire‐9 (PHQ‐9)*, developed by Kroenke, Spitzer, and Williams [22] and adapted and validated in Brazil by Santos et al. [23]. This instrument includes 9 items based on DSM‐IV criteria. Total scores range from 0 to 27 and were categorized as follows: 0–4 (no depressive symptoms), 5–9 (mild depressive symptoms), 10–14 (moderate depressive symptoms), 15–19 (moderately severe depressive symptoms), and ≥ 20 (severe depressive symptoms).

#### 2.4.2 Independent Variables (Exposures)

- **Depression (in Model 1)**: Used as a predictor of social frailty in regression analyses.
- **Social Frailty (in Model 2)**: Used as a predictor of depression in regression analyses.
- **Social Support**: Measured by the *Medical Outcomes Study Social Support Scale (MOS)*, developed by Sherbourne and Stewart [24] and validated in Brazil by Andrade [25], was adopted. This scale consists of 19 items divided into five dimensions of social support: material, affective, emotional, informational, and positive social interaction. The score for this instrument is obtained by calculating scores for each domain, ranging from 20 to 100 points, with higher scores indicating higher levels of social support [24].

### 2.5 Covariates and Potential Confounders

- **Sociodemographic and Economic Factors**:
  ○ Sex (male/female)
  ○ Age (continuous)
  ○ Education (years)
  ○ Marital status (with/without partner)
  ○ Income (monthly household income, categorized in minimum wage brackets)
- **Clinical Variables (Health Conditions)**:
  ○ Duration of treatment (in years)
  ○ Medication use (number of medications)
  ○ Transplant‐related data (time since transplant)

### 2.6 Statistical Analysis

Data analysis was conducted using SPSS software version 29. No missing data were observed in the main variables analyzed. Descriptive statistics, including mean, median, and standard deviation, were calculated. The Kolmogorov–Smirnov test was used to assess data normality. As the data did not meet the assumptions of normal distribution, non‐parametric tests were applied: the Mann–Whitney U test for comparisons between two groups and the Chi‐square test for comparisons involving three or more groups.

Two multiple linear regression (MLR) models were developed to analyze continuous outcomes. The first model explored the association between social frailty and independent variables (such as the HD/TX group and MOS social support scores). The second model examined depression scores as the dependent variable, with social frailty as one of the predictors.

The MLR models were estimated using the Ordinary Least Squares (OLS) method in the *car* package [26] within the Jamovi 2.2.2 software. Model diagnostics included tests for residual normality (Kolmogorov–Smirnov), heteroscedasticity (Breusch–Pagan), and model specification (Durbin–Watson). Model fit was assessed using R2, Root Mean Square Error (RMSE), Akaike Information Criterion (AIC), and Bayesian Information Criterion (BIC). Statistical significance was set at *p* ≤ 0.05.

## 3 Results

The study included 284 patients with chronic kidney disease (CKD), divided into two groups: HD (*n* = 80) and TX (*n* = 204). The **s**ociodemographic and clinical characteristics of the participants are presented in Table 1. HD patients were significantly older on average (59.63 ± 15.14 years) compared to TX patients (43.77 ± 12.65 years; *p* < 0.001). The average level of education was also significantly lower in the HD group (6.66 ± 3.91 years) compared to the TX group (12.08 ± 4.27 years; *p* < 0.001). Regarding medication use, TX patients took more medications on average (8.05 ± 4.41 vs. 6.34 ± 5.14; *p* = 0.005). No significant differences were found between the groups in terms of treatment duration, sex, or marital status (Table 1).

**TABLE 1:** Patient characteristics by treatment group.

	HD (*n* = 80)	TX (*n* = 204)	*p*‐valor^a^
Age (years)	59.63 ± 15.14	43.77 ± 12.65	0.001
Level education (years)	6.66 ± 3.91	12.08 ± 4.27	< 0.001
Number of medications	6.34 ± 5.14	8.05 ± 4.41	0.005
Treatment duration (years)	4.55 ± 5.7	4.09 ± 3.52	0.150
Sex (%)	55% female/45% male	51% female/49% male	0.598
Marital status with a fixed partner (%)	65%	69.1%	0.572

^a^ Test Mann–Whitney.

Regarding social frailty, a higher prevalence was observed in HD patients (51.2%) compared to TX patients (24%), with a statistically significant difference (*p* < 0.001). Conversely, TX patients had a higher proportion of non‐frail individuals (24% vs. 3.8%). The majority of participants in both groups exhibited depressive symptoms ranging from mild to severe. However, there were no statistically significant differences between the groups regarding the different levels of symptom severity (Table 2).

**TABLE 2:** Distribution of social frailty, depression, and social support.

	HD (*n* = 80)	TX (*n* = 204)	*p*‐valor^*^
Social frailty (%)			
Non frail	3.8	24	< 0.001
Pre frail	45	45	
Frail	51.2	24	
Depression (%)			
Without depression	16.3	38.7	0.076
Mild depression	22.5	20.1	
Moderate depression	12.5	15.2	
Moderately severe depression	20.0	10.8	
Severe depression	28.7	15.2	
Social support			
Material support	81.71 ± 24.19	38.83 ± 10.78	< 0.001
Affective support	79.92 ± 23.28	83.40 ± 20.38	0.707
Emotional support	76.81 ± 25.11	73.40 ± 23.86	0.028
Positive social interaction support	74.31 ± 26.51	76.55 ± 22.35	0.293
Information support	76.56 ± 24.10	74.49 ± 22.83	0.067

^*^ *p* < 0.05.

Regarding **social support**, HD patients reported significantly **higher material support** (81.71 ± 24.19) compared to TX patients (38.83 ± 10.78; *p* < 0.001). HD patients also reported higher **emotional support** (76.81 ± 25.11) compared to TX patients (73.40 ± 23.86), with a significant difference (*p* = 0.028). However, no significant differences were found in **positive social interaction support**, **information support** and **affective support** between the groups (*p*‐values of 0.293, 0.707 and 0.067, respectively) (Table 2).

The MLR analysis indicated that patients in the TX group exhibited significantly lower levels of social frailty, with a moderate negative effect (β = −0.60; *p* < 0.001). Higher levels of social support were also associated with lower levels of frailty, demonstrating a moderate negative effect (β = −0.40; *p* < 0.001). Other variables associated with increased frailty included the number of medications, with a weak effect (β = 0.11; *p* = 0.022), and low income: individuals earning up to one minimum wage showed greater frailty, with a moderate effect (β = 0.51; *p* < 0.001), while those earning between two and three minimum wages exhibited a weak effect (β = 0.42; *p* = 0.002). Furthermore, fewer years of education were associated with higher levels of social frailty, showing a weak but significant negative effect (β = −0.13; *p* = 0.029). It is important to note that no significant multicollinearity was detected among the variables, as all variance inflation factors (VIFs) were below 2, except for income ≤ one minimum wage (VIF = 1.95) (Table 3).

**TABLE 3:** Multiple linear regression for the dependent variable social frailty.

Variable	Model				
	Coef.^a^	SE	β	VIF	*p*
Intercept	4.18	0.373	—	—	< 0.001
TX‐HD Group	−0.82	0.177	−0.60	1.40	< 0.001
Social Support	−0.03	0.004	−0.40	1.02	< 0.001
Number of Medications	0.03	0.015	0.11	1.04	0.022
Years of Education	−0.04	0.070	−0.13	1.45	0.029
Income ≤ one minimum wage	0.70	0.194	0.51	1.95	< 0.001
Income two or three minimum wages	0.57	0.184	0.42	1.84	0.002

Abbreviations: β, estimated value or slope coefficient in the regression line; SE, standard error of beta; VIF, variance inflation factors.

^a^ Coefficient of determination.

In the RLM analysis for depression, social frailty emerged as the strongest predictor, with a moderate effect (β = 0.60; *p* < 0.001), indicating that higher levels of social frailty were significantly associated with more severe depressive symptoms. Belonging to the TX group was associated with lower depression scores compared to the HD group, with a weak negative effect (β = −0.31; *p* = 0.012). Male sex was also linked to lower levels of depression, showing a weak negative effect (β = −0.29; *p* = 0.003). Conversely, a higher number of years of education was modestly associated with slightly higher depression levels, with a weak positive effect (β = 0.14; *p* = 0.013) (Table 4).

**TABLE 4:** Multiple linear regression for the dependent variable depression.

Variable	Model				
	Coef.^a^	SE	β	VIF	*p*
Intercept	4.60	1.40	—	—	0.001
Grupo TX‐HD	−2.63	1.04	−0.31	1.40	0.012
Social Frailty	3.40	0.31	0.60	1.15	< 0.001
Male sex	−2.41	0.80	−0.29	1.02	0.003
Years of Education	0.24	0.10	0.14	1.38	0.013

Abbreviations: β, estimated value or slope coefficient in the regression line; SE, standard error of beta; VIF, variance inflation factors.

^a^ Coefficient of determination.

## 4 Discussion

The results highlight important relationships between clinical condition, social support, socioeconomic factors, and depression, contributing to a multidimensional understanding of care in CKD. HD patients exhibited a higher prevalence of social frailty (51.2%) compared to TX patients (24%). Social support was inversely associated with social frailty (β = −0.40; *p* < 0.001), while social frailty emerged as the primary predictor of depressive symptoms (β = 0.60; *p* < 0.001).

Regarding social frailty, Usui et al. [3] report in their study a prevalence of social frailty in elderly hemodialysis patients of 59.5%, a value marginally higher than the 51.2% found in the present study. Despite this numerical difference, both studies indicate a high prevalence of social frailty among hemodialysis patients, supporting the hypothesis that continuous HD treatment, along with chronic complications associated with kidney disease, can substantially impact the worsening of social frailty conditions.

- Factors Associated With Social Frailty

Patients in the TX group exhibited significantly lower levels of social frailty compared to those undergoing HD, with a moderate effect size (β = −0.60; *p* < 0.001). This finding supports the hypothesis that HD, being more frequent, physically demanding, and restrictive, may exacerbate social frailty. The study by Usui et al. [3] aligns with these results, reporting a high prevalence of social frailty among HD patients, attributed to the physical and psychological burden of continuous treatment, social isolation, and limitations in daily activities.

Another factor strongly associated with lower levels of social frailty was social support, which demonstrated a moderate negative effect (β = −0.40; *p* < 0.001). This finding is consistent with the literature, as evidenced by the systematic review of Hamid et al. [7], which highlights the crucial role of social support in predicting not only social frailty but also a range of adverse health outcomes, including mortality, physical difficulties, and disability. Hamid et al. [7] suggest that social networks, social activities, and social support are fundamental to the well‐being of patients, supporting the idea that higher social support can mitigate frailty, as observed in the present study.

Additionally, a higher number of medications was positively associated with social frailty (β = 0.11; *p* = 0.022), suggesting that more complex therapeutic regimens may compromise patients' autonomy and their ability to engage socially. Furthermore, low income and years of education emerged as one of the predictors of social frailty, with moderate to weak effects depending on the income bracket. This finding underscores the crucial role of socioeconomic conditions in shaping social frailty among patients with CKD. Hamid et al. [7] highlighted how financial struggles and social roles contribute to frailty, with medication management potentially worsening these challenges, particularly for CKD patients undergoing prolonged, complex treatments.

II Social Frailty as a Predictor of Depressive Symptoms

The depression showed that social frailty was the strongest predictor of depressive symptoms (β = 0.60; *p* < 0.001), reinforcing the direct relationship between social isolation, restrictions in social interactions, and psychological distress. These findings are consistent with those of Bahall et al. [27], who associated fragile social networks, frequent hospitalizations, and isolation with a higher prevalence of depressive symptoms in patients with CKD.

In addition to social frailty, belonging to the transplant (TX) group was also associated with lower levels of depression (β = −0.31; *p* = 0.012), which reinforces the psychosocial benefits of renal transplantation already observed in previous studies, such as that of Tian et al. [28], who reported a lower prevalence of depression among transplant recipients compared to dialysis patients. Male sex was also associated with lower depression scores (β = −0.29; *p* = 0.003), a result consistent with some literature findings that report higher levels of depressive symptoms among women with CKD [29, 30].

In the present study, higher educational attainment was weakly but significantly associated with more intense depressive symptoms (β = 0.14; *p* = 0.013). Although this finding may seem counterintuitive, it could reflect a heightened awareness of disease severity, reduced tolerance for the limitations imposed by CKD, or higher expectations regarding quality of life. A cross‐sectional study investigating the prevalence of depression in pre‐dialysis CKD patients corroborates the complexity of this issue by identifying marital status (single) and advanced CKD stage as significant predictors of depression, with higher prevalence observed among unmarried individuals and those in more severe stages of the disease [31].

The findings of this study reinforce the importance of a multidimensional approach to the care of patients with chronic kidney disease (CKD), extending beyond clinical interventions to include psychosocial support. The identification of social frailty as a central predictor of depression highlights the need for continuous monitoring of this dimension and the implementation of strategies aimed at strengthening support networks, particularly among patients undergoing hemodialysis (HD).

Interventions targeting material, emotional, and informational support, as well as public policies aimed at reducing economic vulnerability (such as income assistance and improved access to medications) may help mitigate the negative effects of social frailty. Furthermore, the development of educational and community support programs could enhance patients' emotional well‐being and social integration.

III Limitations and Future Directions

Among the limitations of the present study, the cross‐sectional design stands out, as it precludes causal inference between the variables analyzed. Additionally, the use of self‐reported instruments may be subject to recall bias or social desirability bias, particularly concerning the reporting of depressive symptoms and social support. Specific comorbidities, which may influence both social frailty and levels of depression, were also not considered.

Moreover, the study was conducted during the post‐COVID‐19 pandemic period, which may have affected participants' levels of social interaction and mental health. The sample, composed of patients from a single center, also limits the generalizability of the findings to other populations and sociocultural contexts.

Future research should adopt longitudinal designs to explore trajectories of social frailty and mental health over time, as well as include multiple centers and more diverse samples. Qualitative studies may also contribute to a deeper understanding of the experiences of social support and social frailty among patients with CKD.

## 5 Conclusion

The findings of this study highlight social frailty as the primary predictor of depressive symptoms in patients with CKD. Patients in the TX group exhibited lower levels of social frailty and depression, suggesting a potential protective effect of this treatment modality. Additionally, lower income levels and a higher number of medications were associated with increased social frailty, while male sex was linked to fewer depressive symptoms. These results underscore the importance of interventions aimed at reducing social frailty and enhancing social support as relevant strategies for the prevention and management of depression in this population.

## Author Contributions

The concept and design of the study were carried out by Diana Gabriela Mendes dos Santos, Marisa Silvana Zazzetta, and Fabiana de Souza Orlandi. The acquisition of subjects and/or data was prepared by Diana Gabriela Mendes dos Santos, Luma Geraigire, Cleanderson Costa da Silva, Layana Giselly Silva Ferreira, and Ana Carolina de Souza Dinardi. Data analysis and interpretation were performed by Diana Gabriela Mendes dos Santos, Fabiana de Souza Orlandi, and Luma Geraigire. The manuscript preparation was done by Diana Gabriela Mendes dos Santos, Luma Geraigire, Cleanderson Costa da Silva, Layana Giselly Silva Ferreira, and Ana Carolina de Souza Dinardi.

## Funding

This work was supported by Coordenação de Aperfeiçoamento de Pessoal de Nível Superior, 88887.484551/2020‐00 and Fundação de Amparo à Pesquisa do Estado de São Paulo, 2018/22186‐2, 2022/14142‐0.

## Disclosure

The authors have nothing to report.

## Data Availability

The data that support the findings of this study are available on request from the corresponding author. The data are not publicly available due to privacy or ethical restrictions.
